# Pain assessment following open hemorrhoidectomy under local anesthesia versus saddle block: a multicenter randomized controlled trial

**DOI:** 10.1186/s12893-023-02030-6

**Published:** 2023-05-12

**Authors:** Franck Katembo Sikakulya, Robinson Ssebuufu, Xaviour Francis Okedi, Moris Baluku, Herman Lule, Sonye Magugu Kiyaka, Patrick Kyamanywa

**Affiliations:** 1 Faculty of Clinical Medicine and Dentistry, Department of Surgery, Kampala International University Western Campus, PO.Box 70, Ishaka-Bushenyi, Uganda; 2 Faculty of Medicine, Université Catholique du Graben, Butembo, Democratic Republic of the Congo; 3 Uganda Medical and Dental Practitioners Council, Kampala, Uganda; 4 Department of Anesthesia and Critical Care, Kabale University, Kabale, Uganda; 5 Department of Surgery, Kiryandongo Hospital, Kiryandongo, Uganda; 6 Uganda Martyrs University, Nkozi, Uganda

**Keywords:** Pain, Open hemorrhoidectomy, Local anesthesia, Saddle block, Uganda

## Abstract

**Background:**

There is disparity in evidence on pain assessment post open hemorrhoidectomy (OH) using local anesthesia and its use in developing countries compared to developed countries. Therefore, we conducted this study to assess the occurrence of postoperative pain following open hemorrhoidectomy under local anesthesia versus saddle block for uncomplicated 3^rd^ or 4^th^ degree hemorrhoids.

**Methods:**

This was a prospective equivalence randomized, double blind controlled trial conducted from December 2021 to May 2022 among patients with primary uncomplicated 3^rd^ or 4^th^ degree hemorrhoids. Pain severity was assessed at 2, 4 and 6 h post open hemorrhoidectomy using visual analogue scale (VAS). Data was analysed using SPSS version 26 at a *p* < 0.05 as statically significant using visual analogue scale (VAS).

**Results:**

We recruited 58 participants in this study who underwent open hemorrhoidectomy under local anesthesia or saddle block (29 participants per group). The sex ratio was of 1.15 of female to male and a mean age of 39 ± 13. VAS was found to be different at 2 h post OH compare to other time of pain assessment but not statically significant by area under the cover (AUC) (95% CI = 486–0.773: AUC = 0.63; *p* = 0.09) with a none significance by Kruskal–Wallis’s test (p:0.925).

**Conclusion:**

Local anesthesia was found to be having a similar pain severity occurrence in post operative period among patients undergoing open hemorrhoidectomy for primary uncomplicated 3^rd^ or 4^th^ degree hemorrhoids. Close monitoring of pain in postoperative period is mandatory especially at 2 h to assess need of analgesia.

**Trial registration:**

Pan African Clinical Trials Registry, PACTR202110667430356. Registered on 8^th^ October, 2021.

## Background

Hemorrhoids is the most prevalent anal disease with various management options as per the choice of patients and his surgeon which range from conservative treatment (dietary and sclerotherapy) to surgical methods like band ligation and excision according to the grade of hemorrhoids [1, 3]. Conventional hemorrhoidectomy includes Milligan-Morgan (open) and Ferguson–Heaton (closed) techniques and for many years these have been approved as the bench mark for surgical management of hemorrhoidal disease globally [4] with Milligan-Morgan as the goal standard [16].

Anorectal surgery requires adequate anesthesia due to its multiple nerve supply [9]. Painful stimuli can be blocked either with regional or general anesthesia (GA), usually with muscle relaxants [8]. Different studies have suggested that switching from GA or regional anesthesia like saddle block and or spinal anesthesia to local infiltration or perianal block (PAB) may prove advantageous for proctologic surgeries [17].

Controversial results have been published before as to the occurrence of pain following open hemorrhoidectomy under local anesthesia versus spinal anesthesia or general anesthesia. In the United Kingdom and in Brazil, studies found a similar mean pain scores in 10 days follow-up [8, 13] yet in India and in Egypt, the mean rate of pain was seen to be lower post open hemorrhoidectomy under local anesthesia (LA) compared to that done under SA [1, 2, 19].

Despite the use of different anesthetic techniques, postoperative pain remains a major problem in up to 40% of patients during postoperative period [7, 16]. In Uganda, little has been documented about the occurrence of pain in postoperative period among patients who undergo open hemorrhoidectomy using local anesthesia versus saddle block. Therefore, our aim was to assess the occurrence of postoperative pain following open hemorrhoidectomy under local anesthesia versus saddle block in three majors hospitals in rural western Uganda in order to provide evidence based data for switching from use of GA or regional anesthesia in a country where only 0.05 anesthetic providers per 100,000 population are listed [10].

## Methods

### Study design and site

This study was a prospective equivalence double blind randomized controlled trial (RCT) conducted in the department of surgery at Kampala International University Teaching Hospital (KIU-TH), Kitagata and Ishaka Adventist Hospitals in Western Uganda. Patients were randomly assigned to either the local anesthesia group (A) or the saddle block group (B). Patients and outcome assessors were blinded about the anesthesia used.

### Study population, inclusion criteria

All patients admitted to the surgical ward for elective hemorrhoidectomy during the study period were considered as our population of the study. Patients aged 18–65 years with uncomplicated 3^rd^ or 4^th^ degree hemorrhoids and classified by American Society of Anesthesiologist (ASA) as I and II were included in this study.

### Sample size determination

We hypothesized that the mean post-operative pain scores after hemorrhoidectomy using local anesthesia was not different from that done under saddle block. Using the formula for an equivalence design in randomized control trials by Zhong [20] and according to a similar randomized control trial that compared open hemorrhoidectomy under local anesthesia versus supine anesthesia at the Korle Bu Teaching Hospital in Ghana [5], difference in mean pain scores between the 2 groups δ = (0.73,pooled standard deviation in mean pain scores S = 1.9 (average of 2.356 for local anesthesia and 1.479 for supine anesthesia), an estimated sample size of 104 participants per group was obtained. Since at the time of the proposed study, nothing was known about the mean pain score following OH under local anesthesia and the real difference in clinical outcome between the two techniques in Uganda, the sample size was adjusted using Slovin’s formula as detailed by Ellen [17] based on the hospital records, a sample size of 29 participants in each arm was obtained after an addition of 10% in each arm to compensate for loss to follow and non-responsiveness found.

### Study randomization

The participants were allocated randomly to either the Local Anesthesia group (Group A) or the Saddle Block group (Group B) by using envelope allocation concealment whereby 58 envelops were obtained and inside them half of the envelopes was containing a chit with letter A while the other half was having a chit with letter B signifying local anesthesia and Saddle block respectively. This was then kept in a safe box whereby the participants were told to pick one envelope from the box at the time of surgery.

### Participant recruitment and study procedure

The patients admitted for elective hemorrhoidectomy were undergoing pre-anesthetic evaluation the day before surgery and were given fasting guidelines including 6 h for solid foods and 2 h to anesthesia for clear fluids like water. All patients were receiving prophylactic antibiotics (Intra-venous 500 mg of metronidazole) 1 h before incision [12]. All patients scheduled for surgery were consented and admitted a day prior to surgery, prepared and included on the theatre program in order to maintain continuity of care. The participants were allocated using envelope cancealment technique and their identification parameters were recorded while consenting to participate to the study. Intraoperatively, patients underwent open hemorrhoidectomy in lithotomy position, using a mixture of bupivacaine 0.5% at a maximum safe dose of 2 mg/kg with adrenaline (1:200,000) for local anesthesia group or 1.5 ml of 0.5% bupivacaine for saddle block group following the technique of Kavitha Jinjil [11]. Postoperatively, the occurrence of pain following open hemorrhoidectomy was determined in both groups using the Visual analogue Scale (VAS). The VAS was used in interval of 2, 4, 6 h and 7 days postoperatively (the lower end of the scale labeled “0” means no pain while upper end of the scale “10” signifies worst imaginable pain). Analgesia was given according to the Visual analogue Scale (VAS) once its rated more than 4 by the patient at 2, 4 and 6 h for all patients irrespective of the type of anesthesia and all participants were discharged based on the post-anesthesia discharge scoring system (PADS) for determining home-readiness whereby patient was judged fit for discharge when his score was ≥ 9 [15] and follow up on telephone call. Participants were reassessed using the VAS on the 7^th^ day postoperative. Diclofenac sodium 100 mg oral 8 hourly for five days was considered as rescue analgesia postoperatively. All patients were receiving tablet of Metronidazole 400 mg 8 hourly for the five post-operative days. Follow-up research assistants different from the recruiting team were collecting outcome data from patients at stipulated time and entering it into Microsoft Excel sheet up to the 7^th^ day postoperative. Patients were called back for review and follow-up assessment. Those who were not be able to come back to the hospital after discharge were interviewed on a telephone call.

### Data processing and analysis plan

Data was statistically analysed using IBM Statistics SPSS for Windows 23.0. The primary outcome analysed all randomized patients (on an intent-to-treat (ITT) basis) and per-protocol (PP) population. The mean pain scores at rest and their standard deviations were computed and compared using one-way ANOVA tests. VAS scale was stratified as ordinal and the significance in difference in mean scores between local and saddle groups determined by Kruskal–Wallis test at 95% Confidence interval, regarding *p* < 0.05 as statistically significant.

Furthermore, the mean pain scores were further analyzed for significant differences in area under the curve (AUC) for VAS (2-sample t-test). Cross tabulation was performed and odds ratio computed for each technique of open hemorrhoidectomy. Chi-square (x^2^) test of significance was used in order to compare proportions between qualitative parameters.

### Ethical considerations

The research topic was approved by the department of surgery, the faculty of medicine and dentistry, the directorate of postgraduate studies and research and Kampala International University Research Ethics Committee (KIU-REC) under the number KIU-REC-2021–24. After approval by the KIU-REC, the trial was registered with Pan African Clinical Trials Registry under the number PACTR 202,110,667,430,356 on 8^th^ October, 2021. The approval letter was presented to the hospital administration of KIUTH, Kitagata and Ishaka Adventist Hospitals for permission to proceed with the data collection for the study at the study site. Noted that the protocol of this trial was published by BMC trials under the link: https://trialsjournal.biomedcentral.com/articles/10.1186/s13063-022-06636-8.

## Results

### Over view of the result finding

**Fig. 1:**
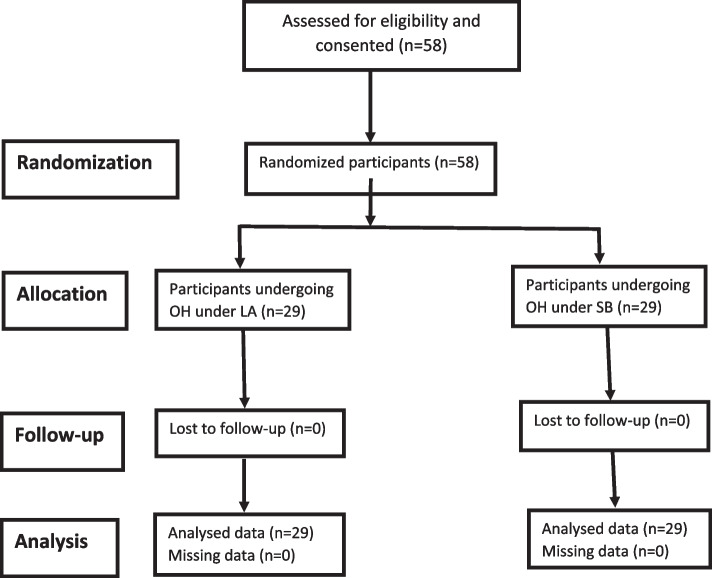
Consolidated standards of reporting trials flow diagram

The Consolidated Standards of Reporting Trials (CONSORT) diagram for patients’ recruitment is shown in the figure below. All the 58 patients recruited consecutively were randomized and followed up to day 7 post OH. The analysis did not have any missing data (Fig. 1).

### Demographics data among patients undergoing OH under LA group versus SB group

Among 58 patients who underwent surgery during this study, the mean age was of 39 ± 13 with a sex ratio of 1.15 of female to male. Peasant constituted the majority of our data. Most patient were classified as ASA I and had hemorrhoid grade 4 (Table 1).

**Table 1:** Comparison between LA group and SB group according to demographic data

**Demographic data**	**Total (*n* = 58)**	**LA Group** (*n* = 29)	**SB Group** (*n* = 29)	**t/X**^**2#**^	***P*-value**
**Hospital**				2.84^#^	0.24
Kitagata Hospital	29	14	15		
KIUTH	15	10	5		
Ishaka Adventist Hospital	14	5	9		
**Age(years)**				-2.395	**0.02**
Minimum–maximum	18–65	18–57	22–65		
Mean ± SD	39 ± 13	35 ± 11	43 ± 14		
**Sex**				0.624^#^	0.430
Female	31	17	14		
Male	27	12	15		
**Occupation**				4.844^#^	0.184
Peasant	30	16	14		
Business	10	2	8		
Student	9	6	3		
Boda-boda man	9	5	4		
**ASA Classification**				5.472^#^	**0.02**
I	53	29	24		
II	5	0	5		
**Hemorrhoid degree**				**0.74**	0.39
3	6	2	4		
4	52	27	25		

*P* value > 0.05, *t* Independent sample t test, *X*^*2#*^ Chi-square test, *SD* Standard deviation

### Pain scoring among patients undergoing OH under LA versus SB

The mean VAS was significantly higher at 2 h (2.28 ± 1.31) in the group A compare to group B (*p* = 0.05) but not statistically significant at 4, 6 h and on 7 post operative day (*p* > 0.05) (Table 2).

**Table 2:** VAS among patients undergoing OH under LA versus SB

**Variables**	**LA Group** (*n* = 29) Mean ± SD	**SB Group** (*n* = 29) Mean ± SD	**t**	***P*-value**
**Visual analogue Scale (VAS)**				
2 h	2.28 ± 1.31	1.69 ± 0.09	1.969	**0.05**
4 h	2.79 ± 1.11	2.79 ± 1.52	-0.0001	1.00
6 h	1.72 ± 1.03	2.00 ± 1.23	-0.928	0.36
Day 7	0.35 ± 0.55	0.38 ± 0.62	-0.223	0.82

*P* value > 0.05, *t* Independent sample t test, *SD* Standard deviation

### Area under the curve of VAS

**Fig. 2:**
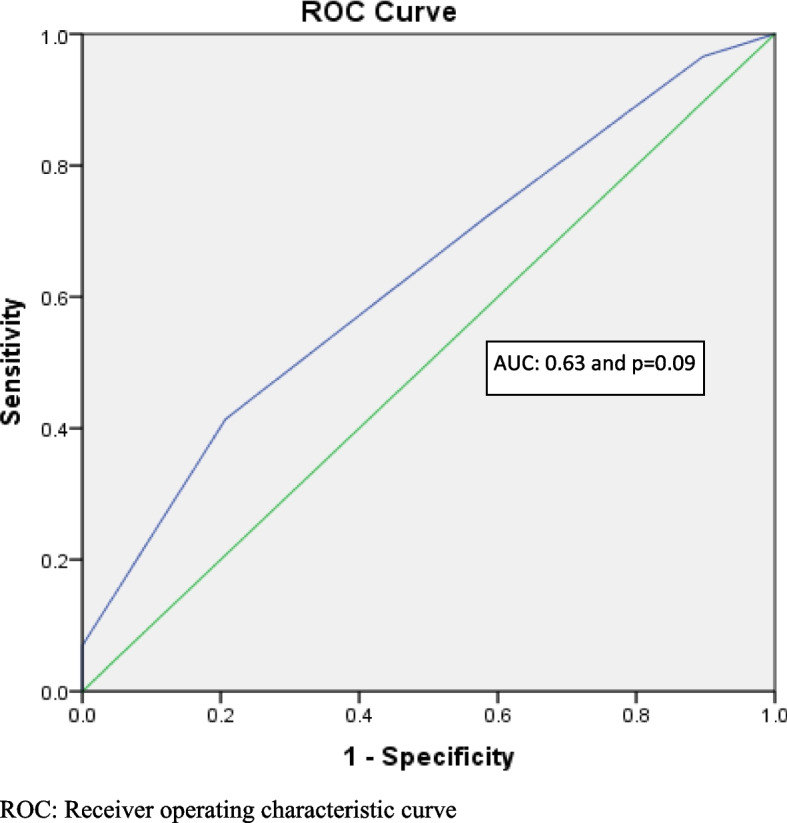
ROC curve showing the diagonal segments of VAS at 2 h post OH under LA vs SB. ROC: Receiver operating characteristic curve

The area under the curve (AUC) on the Receiving Operating Characteristic (ROC) curve below is showing that there is no significant difference in occurrence of pain at 2 h in post-operative period among group A compare to group B (95% CI = 486–0.773: AUC = 0.63; *p* = 0.09) (Table 3 and Fig. 2). The Hypothesis test by Kruskal–Wallis found that the distribution of AUC across the type of anesthesia is not statistically significant (p:0.925) confirming that there is no difference of pain occurrence in post OH in the group A compared to group B.

**Table 3:** AUC by time of VAS assessment comparing the group A to group B

Variables	AUC	95%CI	*p*-value
VAS at 2 h	0.630	0.486–0.773	0.090
VAS at 4 h	0.526	0.373–0.679	0.732
VAS at 6 h	0.431	0.282–0.580	0.367
VAS on Day 7	0.495	0.345–0.645	0.944

*AUC* Area under curve, *95%CI* 95% confident interval, Kruskal–Wallis’s test (p:0.925)

## Discussion

The aim of this trial was to assess the occurrence of postoperative pain following open hemorrhoidectomy under local anesthesia versus open hemorrhoidectomy under saddle block for primary uncomplicated 3^rd^ or 4^th^ degree hemorrhoids in three majors’ hospitals in rural western Uganda. To realize this comparison, we assessed the occurrence of pain in post-operative time at 2, 4, 6 h and on 7^th^ post-operative day in the two groups. Pain occurrence in post-operative period among patients undergoing open hemorrhoidectomy is a common problem reported worldwide and different techniques of anesthesia have been used to carry out the procedure to increase the duration of no pain post-operatively [1, 7].

During our RCT, despite the difference in occurrence of pain assessed with VAS in post OH in the two groups of patients, the AUC has shown no difference in VAS assessment in post OH in both groups. These findings are confirming the null hypothesis of this RCT that no difference between the use of LA versus SB for OH in our area. The above findings are similar to the finding in UK where Kushwaba et al. [13] found out that there was similarity in mean of pain occurrence in post hemorrhoidectomy using LA versus GA over 10 days of follow-up.

However, our result is different to the finding of [1, 2] in India who found that the mean of pain was lower at 90 min and at 6 h post OH. Moreover, in Cairo, Egypt, Younes et al. while comparing open hemorrhoidectomy under local anesthesia than SA found that the mean rate of pain occurrence was significantly lower at 6 h postoperative period [19]. These finding are supporting the reporting of Formiga et al. which stupilated that in anorectal surgery, LA is a common technique that can provide effective pain control and reduce the need for postoperative analgesia [8] and also the finding of Maloku et al. showing that the use of local anesthetic as the sole anesthetic method to control pain after OH [14].

With this data reported worldwide, there is no doubt that the use of local anesthesia is effective for open hemorrhoidectomy and that the occurrence of pain cannot hinder the applicability of this technique for patients with third- or fourth-degree hemorrhoid planned for OH as shown in a newly meta-analysis [18].

### Strengths and limitations of the study

This is the only study assessing the occurrence of post-operative pain following open hemorrhoidectomy under local anesthesia versus open hemorrhoidectomy under saddle block for primary uncomplicated 3^rd^ or 4^th^ degree hemorrhoids in three majors’ hospitals in rural western Uganda. Therefore, it is providing evidence about the use of local anesthesia for uncomplicated 3^rd^ or 4^th^ degree hemorrhoids open surgery in limited setting where few anesthetic providers exist like in Uganda. In addition to that, using VAS and analyzing the result by AUC increase the validity of the findings from this trial. With a sample size of 29 per arm but according to Ellen [6], a sample of around 20 to 50 participants in each arm is sufficient for a validity of a randomized controlled trial study.

## Conclusion

We found that the use of local anesthesia has a similarity of pain occurrence in post operative period among patients undergoing open hemorrhoidectomy for primary uncomplicated 3^rd^ or 4^th^ degree hemorrhoids. A close monitoring of pain in postoperative time is mandatory especially at 2 h to assess need of analgesia. There is a need to update the guideline of management of third- and fourth-degree hemorrhoid in low-income countries like Uganda on the use of local anesthesia for open hemorrhoidectomy for uncomplicated third- and fourth-degree hemorrhoid.

## Data Availability

The datasets used and/or analysed during the current study available from the corresponding author on reasonable request.

## References

[1] Baghel PS, Joleya M, Suryavanshi S (2016). Comparison of open hemorrhoidectomy under local and spinal anesthesia and its practical feasibility at a tertiary care institute. IJSS J Surg.

[2] Bansal H, Jenaw RK, Mandia R, Yadav R (2012). How to do open hemorrhoidectomy under local anesthesia and its comparison with spinal anesthesia. Indian J Surg.

[3] Burch J, Epstein D, Sari AB, Weatherly H, Jayne D, Fox D, Woolacott N, Morgan M (2009). Stapled haemorrhoidopexy for the treatment of haemorrhoids : a systematic review.

[4] Chen J, You J (2010). Paper Alterno Ingles.

[5] Dakubo JCB, Bediako-Bowan AA, Nsaful J, Ampofo A (2015). Day case haemorrhoidectomy under local anaesthesia and conscious sedation. Int J Clin Med.

[6] Ellen S. Slovin's Formula Sampling Techniques" sciencing. com. 2017. 2018. Available at: https://sciencing.com/slovins-formula-sampling-techniques-5475547.html.

[7] Eroglu A, Apan A, Erturk E, Ben-Shlomo I (2015). Comparison of the anesthetic techniques. Sci World J.

[8] Formiga FB, Magi JC, Fernandes B, Boarini LR, Oliveira PD, Vieira RB (2017). Coloproctology the use of local anesthesia and sedation in transanal hemorrhoidal dearterialization with Doppler. J Coloproctol.

[9] Gudaityte J, Marchertiene I, Pavalkis D (2004). Anesthesia for ambulatory anorectal surgery. Medicina (Kaunas).

[10] Hewitt-Smith A, Bulamba F, Ttendo S, Pappenheim K, Walker IA, Smith AF (2018). A mixed-methods evaluation of the association of anaesthetists of great Britain and Ireland Uganda fellowship scheme. Anaesthesia.

[11] Jinjil K, Dwivedi D, Bhatnagar V, Ray RK, Tara S (2018). Perianal block: is it as good as spinal anesthesia for closed hemorrhoidectomies?. Anesth Essays Res.

[12] Kumar A, Aggarwal M, Singla R, Kansal T, Goyal S (2017). Open (Miligan Morgan) haemorrhoidectomy versus stapled haemorrhoidopexy: a comparative Study. British J Med Medic Res.

[13] Kushwaha R, Hutchings W, Davies C, Rao NG (2008). Randomized clinical trial comparing day-care open haemorrhoidectomy under local versus general anaesthesia. Br J Surg.

[14] Maloku H, Gashi Z, Lazovic R, Islami H, Juniku-Shkololli A (2014). Laser hemorrhoidoplasty procedure vs open surgical hemorrhoidectomy: A trial comparing 2 treatments for hemorrhoids of third and fourth degree. Acta Inform Medica.

[15] Marshall SI, Chung F (1999). Discharge criteria and complications after ambulatory surgery. Anesth Analg.

[16] Medina-Gallardo A, Curbelo-Peña Y, De Castro X, Roura-Poch P, Roca-Closa J, De Caralt-Mestres E (2017). Is the severe pain after Milligan-Morgan hemorrhoidectomy still currently remaining a major postoperative problem despite being one of the oldest surgical techniques described? A case series of 117 consecutive patients. Int J Surg Case Rep.

[17] SarangaBharathi R, Sharma V, Dabas AK, Chakladar A (2010). Evidence based switch to perianal block for ano-rectal surgeries. Int J Surg.

[18] Xia W, MacFater HS, MacFater WS, Otutaha BF, Barazanchi AWH, Sammour T, Hill AG (2020). Local anaesthesia alone versus regional or general anaesthesia in excisional haemorrhoidectomy: a systematic review and meta-analysis. World J Surg.

[19] Younes HEA, Metwally YH, El-hussainy AF, Elsayed ME, Ahmad MS (2014). Local anesthesia versus spinal anesthesia for hemorrhoidectomy. AAMJ.

[20] Zhong B (2009). How to calculate sample size in randomized controlled trial?. J Thorac Dis.

